# Correcting for unequal catchability in sex ratio and population size estimates

**DOI:** 10.1371/journal.pone.0184101

**Published:** 2017-08-29

**Authors:** Donald T. McKnight, Day B. Ligon

**Affiliations:** Department of Biology, Missouri State University, Springfield, Missouri, United States of America; Ben-Gurion University of the Negev, ISRAEL

## Abstract

Wildlife populations often exhibit unequal catchability between subgroups such as males and females. This heterogeneity of capture probabilities can bias both population size and sex ratio estimates. Several authors have suggested that this problem can be overcome by treating males and females as separate populations and calculating a population estimate for each of them. However, this suggestion has received little testing, and many researchers do not implement it. Therefore, we used two simulations to test the utility of this method. One simulated a closed population, while the other simulated an open population and used the robust design to calculate population sizes. We tested both simulations with multiple levels of heterogeneity, and we used a third simulation to test several methods for detecting heterogeneity of capture probabilities. We found that treating males and females as separate populations produced more accurate population and sex ratio estimates. The benefits of this method were particularly pronounced for sex ratio estimates. When males and females were included as a single population, the sex ratio estimates became inaccurate when even slight heterogeneity was present, but when males and females were treated separately, the estimates were accurate even when large biases were present. Nevertheless, treating males and females separately reduced precision, and this method may not be appropriate when capture and recapture rates are low. None of the methods for detecting heterogeneity were robust, and we do not recommend that researchers rely on them. Rather, we suggest separating populations by sex, age, or other subgroups whenever sample sizes permit.

## Introduction

A fundamental assumption of many population estimators is that all individuals are equally catchable (or observable), but in nature, truly equal capture probabilities are rarely, if ever, achieved [1]. There are several ways that capture probabilities can vary within a population, and one of the most common occurs when one sex exhibits higher catchability than the other, often because of behavioral differences such as males searching for mates. These sex-specific biases have been observed in a wide range of taxa including: arthropods [2–3], fish [4], amphibians [5], reptiles [6], mammals [7–8], and birds [9].

Heterogeneity of capture probabilities between males and females can adversely affect not only population estimates, but also sex ratio estimates and estimates that rely on accurate sex ratios (e.g., biomass). For example, both Ream and Ream [10] and Gibbons [6] found that there were large differences in the sex ratios of turtles captured by different methods, and relying on any one method resulted in an inaccurate representation of the population structure. Similar artificially biased sex ratios have been reported in other taxa [11–13], and the trapping methods used in those studies caused large deviations from the actual sex ratio.

Several authors have suggested that the impact of sex-specific biases on population estimates could be largely ameliorated by treating males and females as separate populations [14–18]. Although some studies have applied this technique (e.g., Szymanski et al. [19]), there are few formal tests of its consequences in the literature, and this method is often not utilized in studies that would seemingly benefit from it. Two studies that did employ separation of sexes to estimate population sizes [20–21] found that this technique only slightly improved the accuracy of population estimates, and it resulted in low precision. However, both studies involved small populations, and it is not clear if their conclusions are widely applicable.

Another solution to the problem of unequal catchability has been to develop mark-recapture estimators that are specifically designed to deal with heterogeneity in capture probabilities. Many such models are available (reviewed in [22–26]), and ecologists often utilize them rather than blocking their analyses by sex, age, etc. However, even in situations where those estimators produce accurate and precise population estimates, the problem of biased sex ratios remains. In other words, even if a heterogeneity model accounts for the differences in the capture probabilities of males and females and produces a robust population estimate, the sex ratios (which are typically calculated simply as the number of marked males divided by the number of marked females) may still be inaccurate. Additionally, because many species have strong sexual dimorphisms in size, biomass estimates often rely on accurate calculations of the sex ratio. Thus, inaccurate sex ratio estimates may skew other important population parameters such as biomass.

Because of the biases that plague sex ratio estimates, separating sexes may still be a useful technique, even when using heterogeneity models. To our knowledge, only one study has tested the utility of deriving sex ratios from separate population estimates for males and females, and the authors did find a benefit from this method [5]. This result is useful and extremely promising; however, this study only examined one population of one species, so it is difficult to be certain that the result will generally hold true for populations with different levels of trapping heterogeneity.

Here, we used simulations to conduct a thorough test of this method for both population size and sex ratio estimates under a range of conditions, including both open and closed populations. Although this method is intuitive and, based on our observations, appears to be widely recognized among population biologists who are well-versed in the theory behind population estimators, it is often not applied by field ecologists and land managers. Our study aims to bridge that gap between theory and application by focusing on practical applicability rather than theoretical implications. A thorough analysis of the implications of subsetting the data by sex to estimate population size, sex ratio, or both will help to highlight the value and broad applicability of this method. In keeping with that goal, we also used simulations to compare several common tests for equal catchability and determined whether they were adequate for detecting heterogeneous capture probabilities among subgroups.

## Materials and methods

### Simulations separating males and females

We developed two scripts in the program R (version 3.1.1; [27]) to test the concept that the accuracy of population and sex ratio estimates could be improved by treating males and females as separate populations. The first of these simulated a closed population (S1 Script), and the second simulated an open population (S2 Script). Both simulations used the package “Rcapture” (version 1.4–2; [28]) to calculate the population estimates. Both scripts were designed to be versatile, and users can adjust the actual population size, the number of individuals that are captured during each marking period (this can also be set to a range), the sex ratio, and the capture probabilities (i.e., the probability of catching one sex relative to the other). For the open population simulations, it is also possible to set the number of individuals that emigrate and immigrate (these values can be set separately for each sex). Both simulations calculate a population estimate by using all individuals as well as by combining separate estimates for males and females. They also calculate the sex ratio by dividing the number of marked males by the number of marked females and by dividing the calculated male population size by the calculated female population size. For the purposes of this paper, we conducted a series of representative simulations, but the scripts are designed to be easy to use so that readers can conduct additional simulations if desired.

#### Closed populations

The closed population simulator conducts a mark-recapture study with a total of eight sampling periods, and it calculates the population estimates using the “closedp” function. This function calculates population size estimates using the suite of models proposed in Otis et al. [29]. However, for the current study, only the M0 model (no sources of bias) and Mh model (trapping heterogeneity) are relevant because there was neither a time bias (i.e., individuals were equally catchable during each sampling period) or behavioural bias (i.e., being captured did not affect the probability of being captured again). For the sake of having a full range of comparisons available, we programmed the simulation to calculate estimates using both the M0 and Mh models, but we expected the M0 model to be the most accurate for calculations that separate males and females (because separating them removes the heterogeneity), and we expected the Mh model to be the most accurate for estimates that did not separate males and females (except for simulations where males and females were equally catchable, in which case M0 should have been the most accurate). Therefore, we decided a priori that for the comparisons discussed in this paper, we would use the M0 model for estimates that separated males and females, and we would use the Mh model for estimates that did not separate them (except for simulations when no heterogeneity was present).

Within the Mh models, the function can calculate estimates using the models proposed by Chao [30] and Darroch et al. [31], as well as the Poisson model discussed in Rivest and Baillargeon [32]. To determine which Mh model to use for our study, we visually examined the results of 30 simulations that included trapping heterogeneity and returned the full output of the closedp function. The Chao model produced the best result in 20 cases, Darroch in 7, and Poisson in 3. Also, the differences between the results were small, and even when Chao was not the most accurate estimate, it would have been the model that we selected based on BIC values, Pearson residuals, standard errors, and the other descriptive statistics returned by the closedp function. Therefore, we chose the Chao model for our heterogeneity simulations.

We used an actual population size of 300 for all simulations, and we conducted three series of simulations: a series with an even sex ratio and 40 individuals captured during each sampling period, a series with an even sex ratio and 20 individuals captured during each sampling period, and a series with a male biased sex ratio (2:1) and 40 individuals captured during each sampling period. For each series, we simulated 250 populations at each of the following biases: 1, 1.5, 2, 3, and 4 (1 = males and females were equally catchable, 1.5 = males were 1.5 times as likely to be captured as females, 2 = males were twice as likely to be captured as females, etc.).

#### Open populations

The open population simulator conducts a mark-recapture study that is designed to be analysed using the robust design proposed by Pollock [33]. It uses a total of 12 sampling periods, and individuals are able to emigrate and immigrate between periods 3–4, 6–7, and 9–10. Thus, there are four primary sampling periods that are separated by three “open” periods, and there are three secondary sampling periods within each primary period. The individuals that immigrate and emigrate are randomly selected within each sex, and individuals that emigrate can immigrate later and vice versa (immigrants are randomly chosen from a “neighbouring population” that consists of the same number and sex ratio of individuals as the starting population; thus, immigrants are removed from that neighbouring population, and emigrants are added to it). For calculations that include both males and females, the simulation uses the Mh Chao model, and for calculations that separate males and females, it uses the M0 model (see Closed populations).

We conducted six series of simulations, each of which used a starting population size of 300 individuals, an even sex ratio, and 40 individuals captured per sampling period. The six series differed from each other in the number of immigrants and emigrants per open period (series 1 = 5 individuals of each sex immigrated and 5 emigrated during each open period, series 2 = 10 of each sex immigrated and 10 emigrated, series 3 = 20 of each sex immigrated and 20 emigrated, series 4 = 30 of each sex immigrated and 30 emigrated, series 5 = 30 males and 5 females immigrated while 30 males and 5 females emigrated, series 6 = 5 males and 30 females immigrated while 5 males and 30 females emigrated). For each series, we simulated 250 populations at each of the following biases: 1, 1.5, 2, 3, and 4 (1 = males and females were equally catchable, 1.5 = males were 1.5 times as likely to be captured as females, 2 = males were twice as likely to be captured as females, etc.).

### Simulations for tests of equal catchability

We constructed a final R script to test several methods for detecting heterogeneity of capture probabilities (S3 Script). This script conducts a mark-recapture study with eight sampling periods in an identical fashion to the closed population simulator, but instead of calculating population estimates, it runs several published tests for equal catchability: the Chapman test [34], the zero-truncated Poisson test [35], and a chi-square test comparing the number of recaptured and non-recaptured males and females [22,36–37]. To ensure that the calculations in the script were working properly, we tested them on the sample data in Krebs [15].

Additionally, the script tests two methods that we thought were intuitive for examining heterogeneity between subgroups (like males and females). The first of these is simply a Fisher’s exact test comparing the frequencies with which individuals of each sex were captured (i.e., a contingency table with the number of males captured once, number of males captured twice, etc. compared to the number of females captured once, the number captured twice, etc.). The second method uses a Wilcox-Mann-Whitney test to compare the median rank of captures per individual between males and females (i.e., each data point is simply the total number of times a given individual was captured). For this test, the script uses the “wilcox_test” function in the package “coin” (version 1.1–2), because it is capable of dealing with ties [38]. Finally, the script also returns the ratio of marked males to marked females so that it is easy to see how much heterogeneity actually existed.

We conducted two series of simulations with a population size of 300 individuals and an even sex ratio. For the first set, 40 individuals were captured during each capture period, and for the second set, 20 individuals were captured per capture period. For each series, we simulated 250 populations at each of the following biases: 1, 1.5, 2, 3, 4, 5, and 6 (1 = males and females were equally catchable, 1.5 = males were 1.5 times as likely to be captured as females, 2 = males were twice as likely to be captured as females, etc.).

## Results

### Closed populations

**Fig 1:**
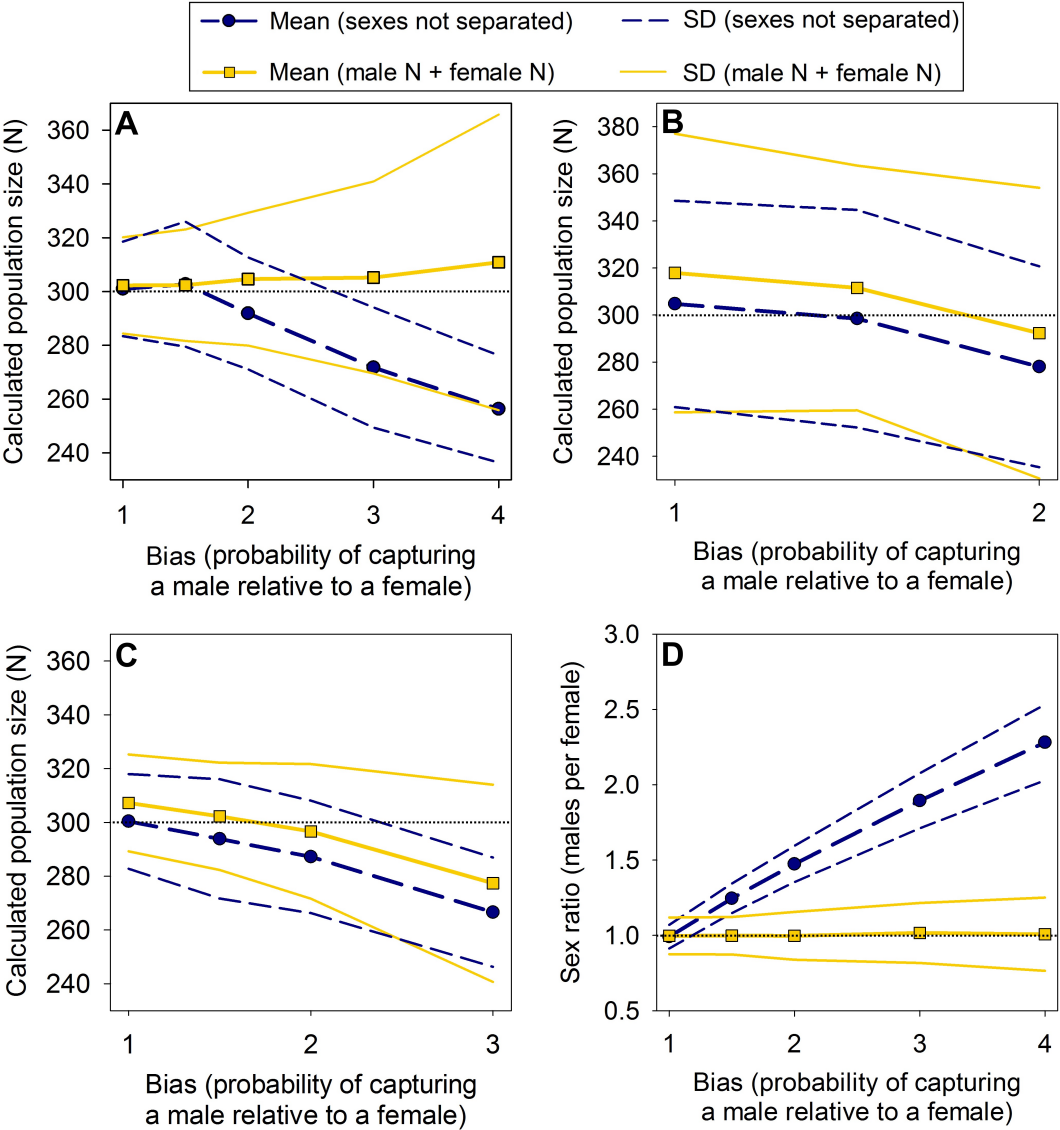
Population estimates and sex ratios from simulations of closed populations. Values are means from 250 simulated populations (“sexes not separated” = estimates were calculated with males and females together as a single population; “male N + female N” = estimates were calculated by treating males and females as separate populations; population size = 300). The horizontal dotted lines show the true values. (A) Captures per sampling period = 40, sex ratio = 1:1 (males:females). (B) Captures per sampling period = 20, sex ratio = 1:1. (C) Captures per sampling period = 40, sex ratio = 2:1. D) Captures per sampling period = 40, sex ratio = 1:1. For B and C, we could not use greater biases, because that resulted in very low capture rates for females and the models would not run (see Discussion).

In many situations, separating males and females resulted in more accurate population estimates. This was especially clear in the simulations where a large number of individuals were captured (Fig 1A). As expected, the benefits of treating males and females as separate populations were greatest when the trapping biases were greatest. In Fig 1A, for example, at both a 3x and 4x male trapping bias, the mean value for estimates that were calculated by separating males and females was very close to the true value; whereas, the mean estimate from the simulations that did not separate sexes was substantially lower than the true population size, and the true value did not fall within one standard deviation of the calculated mean.

The benefits of separating the sexes were especially strong in the sex ratio estimates (Fig 1D). In all of our simulations, the sex ratio that was calculated from treating males and females separately was very close to the true sex ratio, while the sex ratio derived from the number of marked males and females was highly inaccurate even when a relatively small bias was present. This was the case even when only 20 individuals were captured in each recapture period (with no bias, 1.5x male trapping bias, and 2x bias, the mean ratios of marked males to females were 1:1, 1.4:1, and 1.7:1, respectively; whereas the ratios that were calculated by separating males and females were 1.1:1, 1:1, and 1:1, respectively).

This method was not, however, without drawbacks. Although it was usually more accurate, it was less precise for population estimates (precision was similar between methods for sex ratio estimates). Also, when no biases were present, it was generally slightly less accurate than leaving males and females as a single population. Additionally, its accuracy decreased when either few individuals were captured or the sex ratio was male biased (Fig 1B and 1C). However, that result is largely an artifact of our simulation methods (see Discussion).

### Open populations

**Fig 2:**
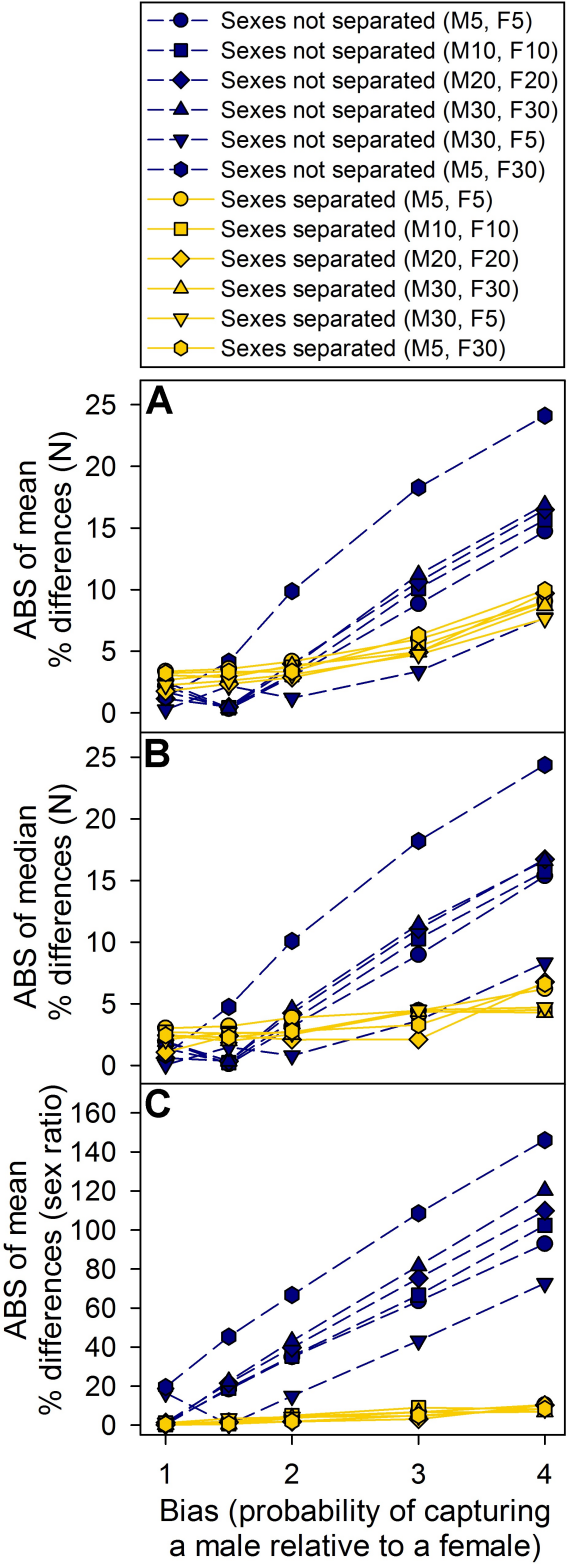
Population and sex ratio estimates from simulations of open populations using the robust model. Sexes not separated = population estimates were calculated with males and females together as a single population and sex ratios were calculated by dividing the number of marked males by the number of marked females; Sexes separated = population and sex ratio estimates were calculated by treating males and females as separate populations; numbers in parentheses are the numbers of emigrants and immigrants per open period [M = males, F = females]; starting populations size = 300; captures per sampling period = 40; starting sex ratio = 1:1 [males:females]). Results are shown as the percent difference between the actual and calculated values because the actual populations sizes and sex ratios changed based on the dispersal settings (the robust model calculates results for any individuals that were present during the entire study period). For ease of comparison, the results are shown as absolute values (ABS); however, keeping males and females together underestimated population sizes and overestimated sex ratios (males relative to females), whereas separating the sexes overestimated the population sizes and slightly underestimated the sex ratios. (A) Mean population estimates. (B) Median population estimates. (C). Mean sex ratio estimates.

The results of the open population simulations were similar to those of the closed populations. Separating males and females generally produced more accurate estimates, but the precision was lower, especially when the trapping bias was high (Fig 2A and 2B). Also, both estimates were still inaccurate when bias was present. Separating the sexes generally overestimated the population size, while keeping them together underestimated the population size. Additionally, separating the sexes when large trapping biases were present sometimes resulted in a right-skewed distribution of simulation results, which can be illustrated by comparing the mean and median results (Fig 2A and 2B). These outliers were generally the result of models that were poorly fit due to a low numbers of captures for one sex, and are largely an artifact of our simulation design (see Discussion). In real populations, model outputs can be used to determine whether or not a model is poorly fit, and these spurious results can be avoided.

The sex ratio estimates were generally much more accurate when males and females were separate; however, they tended to slightly underestimate the true value when the male trapping bias was high. Nevertheless, that slight underestimate was still more accurate than the large overestimate that resulted from simply dividing the number of marked males by the number of marked females (Fig 2C).

### Tests of equal catchability

**Fig 3:**
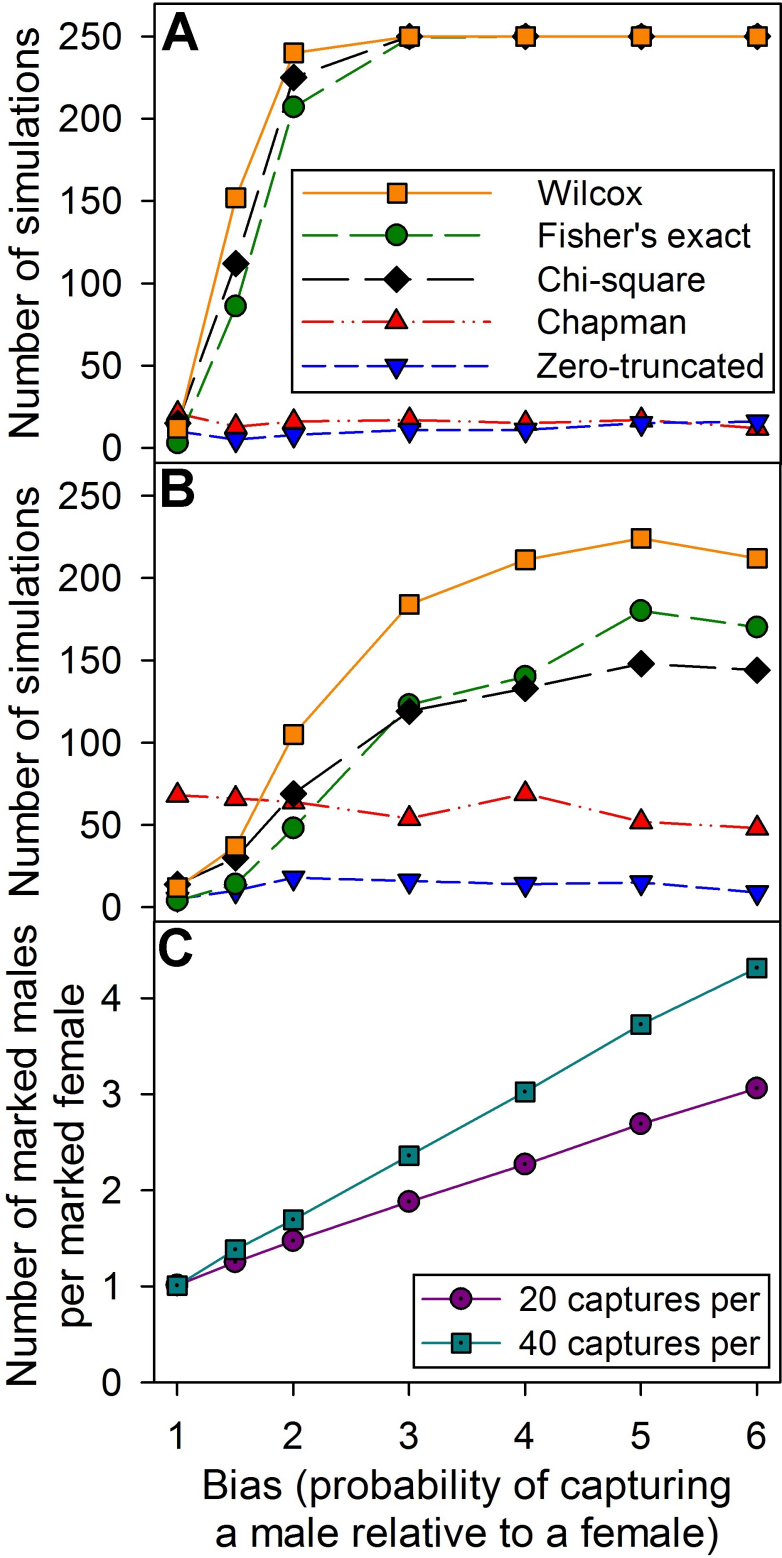
Results of simulations comparing the tests for equal catchability. The following tests were compared: Wilcox-Mann-Whitney *U* (this paper), Fisher’s exact (this paper), chi-square [14,23,36], Chapman [34], and zero-truncated Poisson [35]. (A) The number of tests (out of 250) that gave a significant result when 40 individuals were captured in each sampling period. (B) The number of tests (out of 250) that gave a significant result when 20 individuals were captured in each sampling period. (C) The number of marked males divided by the number of marked females (mean for 250 simulations) despite a 1:1 sex ratio for all individuals (i.e., this shows that high levels of heterogeneity did exist, even when the tests were failing to detect it).

The Chapman and zero-truncated Poisson tests both performed poorly and consistently failed to detect even very large differences in capture probabilities (Fig 3). In contrast, the chi-square test, Fisher’s exact test, and Wilcox-Mann-Whitney test all performed comparatively well when there were large biases and 40 individuals were captured in each sampling period (Fig 3A), but they did not perform well when only 20 individuals were captured (Fig 3B). The Wilcox-Mann-Whitney test performed the best out of the three, but when capture rates were low, it still never detected heterogeneity in more than 90% of samples, even when very large biases were actually present (in the most extreme cases, males were six times as likely to be captured as females). Also, none of the tests were effective at detecting a sampling bias of 1.5, even though these small biases still produced skewed sex ratios (Fig 3C).

## Discussion

### Separating males and females: Population estimates

In general, our results support the conclusion that treating males and females as separate populations is an effective way of dealing with sex-specific heterogeneities of capture probabilities. This method is equally applicable to other subgroups such as age classes. Additionally, we found that the heterogeneity models were typically inadequate for dealing with situations where subgroups had differing capture probabilities, and researchers should not rely entirely on them. Separating males and females is, however, limited by sample size. Population estimators are more precise when a large portion of the population is both captured and recaptured, and splitting males and females effectively cuts the sample size in half [23]. Thus, we expected a loss of precision from this technique.

The fact that the variation increased as the sample sizes decreased explains the inconsistency between our results and the results of Koper and Brooks [20] and McCullough and Hirth [21]. Our results suggest that separating males and females is most effective when sample sizes are fairly large. However, both Koper and Brooks [20] and McCullough and Hirth [21] based their results on small populations, which may explain why they found that treating males and females separately did not adequately improve population estimates.

Throughout our simulations, sample size was continually the main factor that reduced the accuracy and precision of the results. For example, in Fig 1A, the standard deviation increases greatly as the trapping bias increases, but that is a result of sample size. The biases in our simulator are proportional to each other, while the number of individuals captured per capture period is fixed. Thus, if 40 individuals are captured during each sampling period and the bias is 1, then males and females are equally likely to be captured, and, on average, we expect to catch 20 males and 20 females in each sampling period. In contrast, if the bias is set to 4, then we expect to catch four males for every female, so on average, we expect to catch 32 males and only 8 females per sampling period. This resulted in multiple very poor estimates for females, which greatly inflated the standard deviations. Indeed, in many cases (such as the open simulations) we were unable to simulate low overall capture rates or high biases because there were not enough captures of females to obtain a population estimate (e.g., at 20 individuals per sampling period and a bias of four, an average of only four females were captured in each sampling period). Similarly, having a biased sex ratio resulted in very low capture rates for one sex, which reduced the precision of the estimates.

Importantly, the Rcapture package includes very useful outputs such as errors, BIC values, and Pearson residuals that researchers can (and should) use to assess the fit of the chosen model, and for the erroneous female estimates that were produced by low numbers of captures, those outputs showed that the estimates were unreliable. Nevertheless, those estimates were included in our results because our simulation is automated. We chose to include inaccurate estimates rather than having the simulation filter them out because we wanted to give an honest representation of the results, but for actual population estimates, investigators presumably would recognize that those models are inaccurate and would switch to other estimators. In other words, when deciding whether or not to treat males and females as separate populations, researchers should carefully examine the total number of captures and recaptures for each sex to see if those numbers are sufficient for reliable population estimates. Similarly, they should pay careful attention to the model outputs to see if the estimates are reasonable. If the models produce errors or very wide confidence intervals, then researchers may want to leave males and females as a single population rather than treating them separately. In short, researchers should generally separate males and females whenever possible, but in situations where very few individuals of one sex were captured/recaptured, it may not be possible to do so.

### Separating males and females: Sex ratios

The benefits of treating males and females as separate populations were particularly pronounced for sex ratio estimates. This method produced accurate estimates across nearly all of our simulations, even when biases were high and capture rates were low. In contrast, sex ratios that were calculated from simply dividing the number of marked males by the number of marked females were inaccurate anytime that trapping biases were present. Therefore, we strongly recommend that researchers calculate sex ratios by separating males and females and calculating separate population estimates for each group. However, in cases of extreme trapping bias, it may simply be impossible to generate reliable sex ratios.

### Tests for heterogeneity and model selection

We found that two common tests for heterogeneity (Chapman and the zero-truncated Poisson test) are unreliable for detecting heterogeneity between two subgroups. Indeed, these tests are actually designed for detecting the type of heterogeneity that arises from traps modifying behaviour (i.e., being captured changes the probability of being captured again [34–35]), but many researchers use them as a very general test for equal catchability. Based on our results, doing so is inadvisable because these tests will not detect differences in subgroups.

In contrast, both the chi-square method (which is specifically designed for detecting heterogeneity among subgroups [14,23,36]) and the Fisher’s exact test did a good job of detecting heterogeneity when a large number of individuals were captured, but they did not perform as well as simply using a Wilcox-Mann-Whitney test to compare the median ranks of the number of times that individuals were captured. Additionally, none of the tests performed well when few individuals were captured (though the Wilcox-Mann-Whitney test continued to perform the best), and none of them were effective at detecting small differences in capture probabilities, even though those differences still produced biased sex ratios.

Based on these results, if a researcher wants to test their population for equal catchability, it will be necessary to use a test that is specifically designed for detecting differences among subgroups, and we recommend using the Wilcox-Mann-Whitney test (or Kruskal-Wallis if more than two subgroups are present [e.g., males, females, and juveniles]). However, because none of the tests were reliable with small sample sizes or low levels of heterogeneity, we suggest that authors should not rely on them if they fail to detect heterogeneity. Different levels of catchability between males and females is very common, and most real populations probably have at least a small amount of heterogeneity. Therefore, we recommend that authors err on the side of caution and separate their populations into subgroups whenever the sample size permits them to do so.

Finally, it is worth noting that our recommendations only apply to heterogeneity among subgroups. Researchers should still test for other sources of heterogeneity and use the appropriate models to compensate for them. For example, if a population exhibits both heterogeneity between the sexes and a behavioral effect (i.e., being captured changes the probability of being recaptured), then researchers should split the population by sex, and use the appropriate behavior model within each sex.

## Conclusions and recommendations

In summary, our simulations showed that treating males and females as separate populations improved population estimates and, especially, sex ratio estimates. However, for calculating population sizes, this method was less precise, and it was limited by sample size. Therefore, we recommend that researchers use it whenever possible, but in situations where one sex has low capture/recapture rates, it may not be possible to do so. In contrast, this method improved sex ratio estimates in nearly every simulation that involved trapping biases. Therefore, we think that it should be used widely for sex ratio estimates. Additionally, we found that tests for equal catchability are not particularly effective at detecting differences among subgroups. Therefore, we recommend that researchers do not rely on them, and instead simply block their estimates by subgroups whenever possible.

## Supporting information

S1 Script Closed population simulator. A script in R that simulates a mark-recapture study of a closed population and calculates the population size and sex ratio both by grouping males and females together and by treating males and females as separate populations.
(R) Click here for additional data file.

S2 Script Open population simulator. A script in R that simulates a mark-recapture study of an open population and calculates the population size and sex ratio both by grouping males and females together, and by treating males and females as separate populations.
(R) Click here for additional data file.

S3 Script Simulator for detecting unequal catchability. A script in R that simulates a mark-recapture study of a closed population and tests several methods for detecting unequal catchability.
(R) Click here for additional data file.

## References

[1] Carothers AD. Capture-recapture methods applied to a population with known parameters. J Anim Ecol. 1973; 42: 125–146.

[2] Tabashnik BE. Population structure of pierid butterflies. Oecologia 1980; 47: 175–183. doi: 10.1007/BF00346817 28309468 10.1007/BF00346817

[3] Ziegle PE, Frusher SD, Johnson CR, Gardner C. Catchability of the southern rock lobster *Jasus edwardsii*. L. Effects of sex, season and catch history. Mar Freshwater Res. 2003; 53: 1143–1148.

[4] Solmundsson J, Karlsson H, Palsson J. Sexual differences in spawning behavior and catchability of plaice (*Pleuronectes platessa*) west of Iceland. Fish Res. 2003; 61: 57–71.

[5] Pickett EJ, Stockwell MP, Pollard CJ, Garnham JI, Clulow J, Mahony MJ. Estimates of sex ratio require the incorporation of unequal catchability between sexes. Wild Res. 2012; 39: 350–354.

[6] Gibbons JW. Sex ratios and their significance among turtle populations In: Gibbons editor. Life history and ecology of the slider turtle. USA, Smithsonian Institute; 1990 pp 171–182.

[7] Downing RL, Michael ED, Poux RJ. Accuracy of sex and age ratio counts of white-tailed deer. J Wild Manage. 1977; 41: 709–704.

[8] Ogutu JO, Piepho HP, Dublin HT, Reid RS, Bhola N. Application of mark-recapture methods to lions: satisfying assumptions by using covariates to explain heterogeneity. J Zool. 2006; 269: 161–174.

[9] Dwyer TJ, Sepik GF, Derleth EL, McAuley DG. Demographic characteristics of a Maine woodcock population and effects of habitat management. U.S. Fish and Wildlife Service, Fish Wild Res. 1988; 4: 1–29.

[10] Ream C, Ream R. The influence of sampling methods on the estimation of population structure in painted turtles. Am Midl Nat. 1966; 75: 325–338.

[11] King CM. The sex ratio of trapped weasels (*Mustela nivalis*). Mammal Rev. 1975; 5: 1–8.

[12] Topping CJ, Sunderland KD. Limitations to the use of pitfall traps in ecological studies exemplified by a study of spiders in a field of winter wheat. J Appl Ecol. 1992; 29: 485–491.

[13] Dorn NJ, Urgelles R, Trexler JC. Evaluating active and passive sampling methods to quantify crayfish density in a freshwater wetland. J N Am Benthol Soc. 2006; 2006: 346–356.

[14] Begon M. Investigating animal abundance: Capture-recapture for biologists London: Edward Arnold Ltd.; 1979.

[15] Krebs CJ. Ecological methodology USA: Addison-Wesley Educational Publishers; 1999.

[16] Southerwood TRE, Henderson PA. (2000) Ecological methods 3^rd^ ed. Cambridge: Blackwell Science Ltd.; 2000.

[17] Stanford KM, King RB. Growth, survival, and reproduction in a northern Illinois Population of the plains garter snake, *Thamnophis radix*. Copeia 2004; 2004: 465–478.

[18] Pollock KH, Nichols JD, Brownie C, Hines JE. Statistical inference for capture-recapture experiments. Wild Monogr. 1990; 107: 3–97.

[19] Szymanski J, Shuey JA, Oberhauser K. Population structure of the endangered Mitchell’s Satyr, *Neonympha michellii mitchellii* (French): Implications for conservation. Am Midl Nat. 2004; 152: 304–322.

[20] Koper N, Brooks RJ. Population-size estimators and unequal catchability in painted turtles. Can J Zool. 1998; 76: 458–465.

[21] McCullough DR, Hirth DH. Evaluation of the Petersen-Lincoln estimator for a white-tailed deer population. J Wild Manage. 1988; 52: 534–544.

[22] Pollock KH. Mark-recapture models. J Am Stat Assoc. 2000; 95: 293–296.

[23] Seber GAF. The estimation of animal abundance and related parameters 2nd ed. Caldwell, New Jersy: Blackburn Press; 1982.

[24] Seber GAF. A review of estimating animal abundance. Biometrics, 1986; 42: 267–292. 3527287

[25] Seber GAF. A review of estimating animal abundance II. Int Stat Rev. 1992; 60: 129–166.

[26] Schwarz CJ, Seber GAF. A review of estimating animal abundance III. Stat Sci. 1999; 14: 427–456.

[27] R Development Core Team R: A Language and Environment for Statistical Computing. R Foundation for Statistical Computing Vienna 2014; Available from http://www.R-project.org [accessed 1 May 2014]

[28] Baillargeon S, Rivest L-P. Rcapture: loglinear models for capture-recapture in R. J Stat Soft. 2007; 19: 1–31.

[29] Otis DL, Burnham KP, White GC, Anderson DR. Statistical inference from capture data on closed animal populations. Wild Monogr. 1978; 62: 3–135.

[30] Chao A. Estimating the population size for capture-recapture data with unequal catchability. Biometrics 1987; 43: 783–791. 3427163

[31] Darroch SE, Fienberg G, Glonek B, Junker B. A three sample multiple capture-recapture approach to the census population estimation with heterogeneous catchability. J Am Stat Association 1993; 88: 1137–1148. 12155419

[32] Rivest LP, Baillargeon S. Applications and extensions of Chao’s moment estimator for the size of a closed population. Biometrics 2007; 63: 999–1006. doi: 10.1111/j.1541-0420.2007.00779.x 17425635 10.1111/j.1541-0420.2007.00779.x

[33] Pollock KH. A capture-recapture design robust to unequal probability of capture. J Wild Manage. 1982; 46: 757–760.

[34] Chapman DG. Inverse, multiple, and sequential sample censuses. Biometrics, 8: 286–306.

[35] Caughley G. Analysis of Vertebrate Populations. Wiley; 1977.

[36] White EG. Identifying population units that comply with capture-recapture assumptions in an open community of alpine grasshoppers. Res Popul Ecol. 1975; 16: 153–187.

[37] Seber GAF. A note on the multiple-recapture census. Biometrika 1965; 52: 249–59. 14341277

[38] Hothor T, Hornik K, van de Wiel MA, Winell H, Zeileis A. Conditional Inference Procedures in a Permutation Test Framework. CRAN 2015.

